# Effects of Timing of Injectable Trace Mineral Administration on Beef Calf Performance and Health Following Simulated Marketing

**DOI:** 10.3390/ani16101430

**Published:** 2026-05-08

**Authors:** Marie E. Goulais, Miriam A. Snider, Carter Phillips, S. Maggie Justice, Jeremy G. Powell, Cody T. Shelton, Grayson Gourley, R. Cyle Jones, J. Daniel Rivera

**Affiliations:** 1 Department of Animal Science, University of Arkansas System Division of Agriculture, Fayetteville, AR 72701, USA; megoulai@uark.edu (M.E.G.); csp010@uark.edu (C.P.); jerpow@uark.edu (J.G.P.); 2 Southwest Research and Extension Center, University of Arkansas System Division of Agriculture, Hope, AR 71801, USA; msnider@uada.edu (M.A.S.); ctshelto@uada.edu (C.T.S.); ggourley@uada.edu (G.G.); richardj@uada.edu (R.C.J.); 3 Department of Animal Sciences, Auburn University, Auburn, AL 36849, USA; smj0059@auburn.edu

**Keywords:** beef cattle, bovine respiratory syncytial virus, hair cortisol, injectable trace mineral, marketing stress, weaning

## Abstract

The objective of this study was to evaluate the effects of timing of injectable trace mineral (ITM) administration (28 days (d) prior to or at weaning) on performance and health in mixed-sex beef calves (*n* = 115; 224 ± 40 kg). Calves were randomly assigned to one of the following treatments: (1) no ITM (CON), (2) ITM administered 28 d before weaning (PW), or (3) ITM administration at weaning (WEAN). At weaning, calves were transported to a local auction barn, held overnight, and returned the following day; BW, blood, and hair samples were collected prior to and through the receiving period. Data were analyzed using SAS 9.4. Serum Se increased in PW calves following ITM administration (*p* < 0.01). Serum Mn increased in PW and WEAN groups (*p* < 0.01) and PW calves showed increased serum Cu at weaning (*p* < 0.01). Across treatments, calves experienced 6% shrink following weaning and transport, with recovery of BW and intake occurring within 21 d and 8 d, respectively. Despite improved mineral status, no performance benefits were observed during the receiving period, reflecting adequate baseline mineral status and low-stress management conditions, suggesting that ITMs may have limited benefits in well-managed herds.

## Simple Summary

Weaning and transport are stressful events that can negatively impact calf performance and health. Injectable trace minerals (ITMs) are sometimes used to help calves cope with stress, but it is unclear whether the timing of ITM administration influences their effectiveness. This study evaluated whether ITM administration before weaning or at weaning followed by simulated marketing improved calf performance and health compared to calves that did not receive ITM. Calves that received ITM had greater mineral levels in their blood, but this did not lead to better growth, health, or reduced stress after weaning and transport. Regardless of treatment, calves experienced significant weight loss following weaning and transport. Overall, ITM administration did not provide additional benefits under the conditions of this study. These results suggest that in well-managed herds with adequate nutrition, ITMs may not improve calf outcomes during weaning and marketing. This information can help producers decide whether ITM use is likely to be beneficial in their operation.

## 1. Introduction

Weaning represents one of the most stressful events in the life of a calf, often resulting in reduced feed intake and subsequent weight loss [1,2,3]. Following separation from the dam, calves are often transported to a backgrounding or feedlot operation, with 43.1% of United States-based beef producers shipping their calves immediately after weaning [4]. Transportation-induced weight loss represents a major economic challenge in livestock production as the industry operates on a weight-based trading system [5]. During transport, calves may experience prolonged periods without access to feed and water, leading to shrink losses of up to 9% of body weight (BW; [6,7,8,9]). Body weight shrink can be further exacerbated by stress and exposure to novel feed (i.e., unfamiliar receiving diets), which can further reduce voluntary feed intake after arrival and delay weight recovery [10,11]. Additionally, transport-related stress and commingling can suppress immune function, increasing susceptibility to diseases, such as bovine respiratory disease (BRD), and compromising overall health [12,13]. As stress can potentially reduce nutrient supply when physiological demand is at its greatest, proper health management of cattle during this period is critically important [14,15,16,17]. Measures, such as vaccination and parasite control, may reduce the likelihood of disease and improve the overall resilience of the calf following weaning and transportation [1,18].

In addition to proper health management practices, nutritional management following weaning is essential for mitigating stress as the abrupt transition from milk to solid feed can cause nutritional imbalances [1]. Reduced feed intake during this period may contribute to trace mineral (TM) deficiencies, as TM homeostasis is tightly regulated but can be disrupted by stress or rapid growth [15,19,20]. Trace minerals are essential for numerous physiological functions in beef cattle, supporting animal maintenance and the replenishment of body reserves [21] as well as playing critical roles in antioxidant defense and disease resilience, particularly during periods of stress-induced metabolic challenge [22,23]. Additionally, TMs such as copper (Cu), zinc (Zn), selenium (Se), and manganese (Mn) have crucial roles in various biological functions, particularly in immune function and metabolic performance [24,25,26]. These TMs are critical during periods of elevated stress, including weaning, transportation, and commingling with unfamiliar animals [27]. When dietary intake or forage quality do not meet animal requirements, TMs may be supplemented in beef cattle diets using inorganic sources such as salts, chlorides, sulfates, and carbonates. Supplementation of TMs can occur in varying combinations and delivery methods with oral TM supplementation (OTMS) being one of the most common delivery methods [28]. However, producers may opt to use injectable TM (ITM) as a delivery method. Injectable TMs have been shown to be advantageous during high-stress periods when cattle are vulnerable to mineral deficiencies due to reduced feed and mineral intake [29] and increased metabolic demand [20,26,30].

Despite evidence supporting the physiological importance of TMs during stress, there is limited and inconsistent research related to the effects of timing of ITM administration relative to weaning and marketing stress on beef calf performance, immune response, and mineral status [31,32]. This may likely be due to differences in animal health status, baseline mineral reserves, management systems, and stress exposure. Therefore, the objective of this study was to evaluate the effects of ITM administration timing on calf performance, serum mineral status, and health indicators pre- and post-weaning in conjunction with simulated marketing in a well-managed calf herd. It was hypothesized that ITM administration at weaning would mitigate acute physiological responses associated with weaning and marketing stress. Additionally, ITM administration 28 days (d) prior to weaning was hypothesized to improve mineral status prior to stress exposure, enhancing resilience to both weaning and subsequent stress.

## 2. Materials and Methods

### 2.1. Animal Care and Use

All animal procedures were approved by the University of Arkansas System Division of Agriculture Institutional Animal Care and Use Committee (Protocol Number 24057). This study was conducted at the Southwest Research and Extension Center (SWREC) located in Hope, Arkansas, USA from September to December 2024.

### 2.2. Animal Management and Experimental Treatments

One hundred fifteen crossbred (Angus × Brangus) mixed-sex beef calves (average BW = 224 ± 40 kg; age = 202 ± 17.8 d) were utilized in this study. Sample size was based on herd availability. Prior to the beginning of the study, calves remained on bermudagrass (*Cynodon dactylon* (L.) Pers.) pastures with their dams and were managed as a single group. Both dams and calves had ad libitum access to a loose mineral supplement (Forti-Graze, Mineral, Livestock Nutrition Center, Chickasha, OK, USA; Table 1).

All bull calves were surgically castrated within 2 d of birth in accordance with standard management practices. Calves were housed across the Cow/Calf and Stocker Units at the SWREC for the duration of the trial. Prior to the initiation of treatments, calves were blocked by sex (*n* = 59 heifers; *n* = 56 steers) and age with calf ages ranging between 6 and 8 months. Within strata, calves were randomly assigned to one of three treatments consisting of the following: (1) no ITM (CON; *n* = 39), (2) a subcutaneous ITM administered 28 d prior to weaning (PW; *n* = 35), or (3) the same ITM administered on the day of weaning (WEAN; *n* = 39). Mortalities (*n* = 2) were documented and not included in the subsequent statistical analysis.

### 2.3. Calf Performance and Health Response Measures

**Figure 1:**
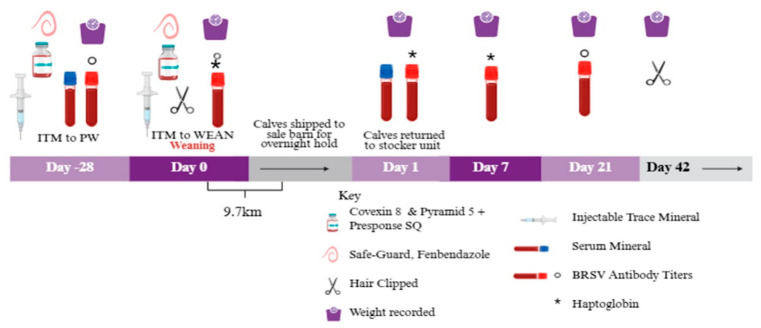
Study sampling schedule for calves across weaning, simulated marketing, and receiving period. Treatments were as follows: (1) no injectable trace mineral (ITM; CON), (2) subcutaneous ITM administration 28 d prior to weaning (PW), or (3) ITM administration on the day of weaning (WEAN). Image created with Biorender.

A timeline of calf processing, treatment application, and sample collection is presented in Figure 1.

#### 2.3.1. Pre-Weaning Period

Twenty-eight days prior to weaning (D-28), all calves were individually weighed. Calves were administered a clostridial vaccine (Covexin 8, Boehringer Ingelheim Animal Health, Duluth, GA, USA) and a modified live respiratory vaccine (Pyramid 5 + Presponse SQ, Boehringer Ingelheim Animal Health), and were dewormed (Safe-Guard, Fenbendazole, Merck Animal Health, Rahway, NJ, USA) according to manufacturer guidelines. Blood samples were collected into serum tubes (10 mL; Serum Clot Activator Tubes, Greiner Bio-One, Monroe, NC, USA) and trace element serum tubes (6 mL; no additive tubes; BD Medical, Franklin Lakes, NJ, USA) via jugular venipuncture using an 18 G needle (BD Medical). All blood samples were stored on ice until processing. Samples were used for baseline determination of bovine respiratory syncytial virus (BRSV) antibody titers and serum mineral concentrations of Cu, Mn, Se, and Zn. Following blood collection, calves assigned to the PW treatment group received a subcutaneous ITM (Multimin 90, Axiota, Fort Collins, CO, USA) according to manufacturer guidelines at a rate of 1 mL/45 kg BW (Table 2).

#### 2.3.2. Weaning, Simulated Marketing, and Receiving Period

All calves were weaned on D0, at which time they were sorted and separated from their respective dams. Calves were individually weighed, received booster vaccinations using the same products administered on D-28, and were dewormed again. Blood samples were collected and stored as previously described for determination of BRSV titers and haptoglobin concentrations. Hair samples for baseline cortisol analysis were collected from the right hip of each calf using electric clippers with a #2 guide comb. Samples were stored individually in plastic bags in a cool dry environment until analysis. Calves assigned to the WEAN treatment group received the same subcutaneous ITM given to the PW treatment group 28 d prior to weaning (Table 2). Calves assigned to the CON treatment group did not receive an injection or carrier solution but were otherwise handled and managed the same as the PW and WEAN groups. Following processing, all calves were transported to a commercial livestock auction barn (Hope Livestock Auction Inc., Hope, AR, USA) located 9.7 km away from the SWREC and housed together overnight (16–20 h) in covered pens with access to water and long-stem hay. This was to simulate normal management practices when calves would be abruptly weaned and shipped to the sale barn for marketing. It should be noted that calves were shipped to the sale barn 2 d prior to the weekly auction. Therefore, while calves were exposed to an unfamiliar environment, they were not subjected to the commingling that would normally occur during a typical marketing situation.

The following day, D1, calves were transported back to the SWREC Stocker Unit and weighed individually. Blood samples were collected to assess serum mineral and haptoglobin concentrations as previously described. Calves were then blocked by BW, sorted by sex and treatment, and housed in 12 outdoor soil-surfaced dry-lot pens (137 m × 37 m). Uniquely colored ear tags correlating to treatment with pen designations were placed in each calf’s ear. Grass in assigned pens had been mowed and due to the season, there was little active forage growth. Each pen was equipped with fenceline feed bunks and automatic waterers. Pens housed 10 calves, providing uniform group sizes across treatment groups. Beyond weaning, individual animal BW was recorded on D7, D21, and D42. Additional blood samples were collected on D7 (haptoglobin) and D21 (BRSV antibody titers). On the final day of the treatment period (D42), hair samples for cortisol analysis were collected and stored as previously described.

For the duration of the 42 d post-weaning receiving period, calves were offered a high-roughage starter ration formulated to be limit-fed at a rate of 2.00% of BW (DM-basis) and to support an average daily gain (ADG) of approximately 0.91 kg/d (Table 3).

Feed allotments were adjusted throughout the study based on periodic BW measurements. Individual animal weights were used to estimate pen-level mean BW, and feed delivery was adjusted accordingly to maintain targeted intake levels. In addition to the starter ration, calves were offered long-stem annual ryegrass (*Lolium multiflorum* Lam.) hay. Hay was weighed prior to feeding and was fed in enclosed hay racks to minimize hay loss. When it was visually estimated that less than 45.5 kg remained, the remaining hay was weighed, sampled for dry matter (DM) and discarded, and a fresh bale weighed and offered. Feed was delivered every morning (08:00 h) via tractor-mounted mixer wagon with calves having ad libitum access to free-choice hay and water. Hay and starter ration nutrient compositions are presented in Table 4.

#### 2.3.3. Animal Health Management

For the duration of the study, calves were monitored daily by trained personnel for signs of illness, injury, or abnormal behavior. Health observations were recorded each morning prior to feeding and any calf exhibiting clinical signs of illness was noted for further evaluation. Suspect animals were physically examined, with rectal temperature recorded using a digital thermometer (M900, GLA, Agricultural Electronics, San Luis Obispo, CA, USA). Calves presenting with a rectal temperature ≥ 39.5 °C in conjunction with clinical signs were classified as morbid and treated according to a pre-established treatment protocol and guidelines [33]. Treatment protocols consisted of an initial antimicrobial administration (Baytril 100, Elanco Animal health, Greenfield, IN, USA) with follow-up monitoring occurring at 24 h intervals. Additionally, during the 42 d post-weaning period, predator activity on four occasions resulted in calves becoming mixed among pens. No injuries or overt signs of distress were noted, and calves were subsequently re-sorted to their designated groups.

### 2.4. Sample Analysis

#### 2.4.1. Forage and Feed Analysis

Annual ryegrass hay bales offered to calves during the 42 d post-weaning period were sampled prior to the start of the study for nutritive analysis. Core samples (5/bale) were collected from round bales to obtain a representative sample for nutrient quality assessment. Hay samples were compiled within a paper bag and weighed. Samples were then dried at 50 °C in a forced air oven for 24 h and weighed again to determine DM content. When a new bale was offered it was sampled in a similar fashion. Feed diet samples were collected weekly then compiled by weigh period. Dried diet and hay samples were shipped to a commercial laboratory for analysis of DM, crude protein (CP), acid detergent fiber (ADF), neutral detergent fiber (aNDF), total digestible nutrients (TDN), net energy of maintenance (NE_m_), and net energy of gain (NE_g_; Dairy One Forage Laboratory, Ithaca, NY, USA).

#### 2.4.2. Blood Metabolite and Serum Mineral Analysis

Blood samples collected on D-28, D0, D1, D7, and D21 were allowed to clot at room temperature for 30 min before centrifugation. Serum samples were centrifuged at 1260× *g* for 10 min at 2 °C within two h of collection, aliquoted, and stored at −20 °C until subsequent laboratory analysis. Samples collected on D-28, D0, and D21 were submitted to the Oklahoma Animal Disease Diagnostic Laboratory using established procedures for evaluation for BRSV serum neutralizing (SN) antibody titers. Serum titers were log_10_ transformed prior to statistical analysis. Negative samples at a dilution of 1:4 were assigned an arbitrary antibody titer of 2 for the calculation of geometric mean titers [34]. Haptoglobin concentrations were determined using a bovine-specific enzyme-linked immunosorbent assay (ELISA; Immunology Consultants Laboratory, Portland, OR, USA), following the manufacturer-recommended protocol with samples analyzed in duplicate. The intra- and inter-assay CVs were 6.9% and 20.7%, respectively.

Serum Cu, Mn, Se, and Zn were quantified and analyzed by inductively coupled plasma mass spectrometry (ICP–MS) at the Michigan State Veterinary Diagnostic Laboratory. Serum sample analysis was conducted following protocols from Wahlen et al. [35]. Briefly, serum samples were diluted 25-fold in a matrix containing EDTA, Triton X-100, ammonium hydroxide, 1-butanol, and internal standards (Sc, Rh, In, and Bi). Elemental concentrations were determined using multi-point calibration curves, and in-house serum pools were included as quality controls.

#### 2.4.3. Hair Cortisol

Hair cortisol analysis was conducted following protocols adapted from Meyer et al. [36] and Moya et al. [37]. Briefly, cortisol concentrations were determined via a coated-tube radioimmunoassay (RIA; MP Biomedicals, Santa An, CA, USA) according to the manufacturer’s instructions. A standard curve was prepared by serially diluting a cortisol standard solution to produce working concentrations ranging from 0.26 ng/mL to 33 ng/mL. Standards were conducted in quadruplicate and samples in duplicate. After incubation, tubes were decanted, dried, and counted using a gamma counter. Cortisol concentrations in unknown samples were calculated based on counts per minute using a standard curve generated from the assay standards. Inter-assay CV was not assessed due to the absence of repeated quality control samples. Intra-assay CV across standard concentrations was 2.02%.

### 2.5. Statistical Analysis

Data were analyzed as a randomized complete block design using the PROC MIXED procedure of SAS 9.4 (SAS Inst. Inc., Cary, NC, USA). Animal performance parameters, serum metabolites (haptoglobin, hair cortisol, and BRSV titers), and serum mineral (Mn and Se) data were analyzed using repeated measures where the animal was the experimental unit and sex and treatment were the fixed effects. For all repeated-measures analyses, time was included as a repeated factor and a first-order autoregressive [AR(1)] covariance structure was used. The Kenward–Roger method was used to estimate denominator degrees of freedom for all models. Treatment (TRT), time, and TRT × time interaction effects were evaluated when appropriate. Haptoglobin and BRSV titers were log_10_ transformed for normality and to meet model assumptions prior to statistical analysis [34,38].

Sex was included as a fixed effect and removed when not considered significant within the model. A time effect between TRT on D-28 for serum Cu was observed; therefore, D-28 was used as a covariate for serum Cu analysis. A significant sex effect was observed for serum Zn; therefore, a sex × time and a sex × TRT interaction was evaluated. Morbidity and mortality were analyzed using the PROC GLIMMIX procedure as nonparametric data with a binomial distribution. For all analyses, least squares means (LSMEANS) were used for treatment comparisons where appropriate. Statistical significance was declared when *p* ≤ 0.05, and tendencies were recognized when 0.05 < *p* ≤ 0.15.

## 3. Results and Discussion

### 3.1. Animal Performance Measures

Weaning method, transportation time, novelty of the receiving environment, commingling, and dietary transition can all negatively influence calf growth performance. Trace minerals, such as Cu, Se, Mn, and Zn, have roles related to metabolism and protein synthesis [32,39], suggesting that ITM administration could potentially affect animal growth and partially negate the impacts of stress. Although calves in this study were exposed to transport and an unfamiliar environment, the absence of commingling with unfamiliar animals may represent a limitation of this model, potentially reducing the overall magnitude of stress compared with typical marketing conditions. Furthermore, brief pen-mixing events due to predator activity may have introduced transient social stress, although no overt effects were observed.

#### 3.1.1. Calf Growth Performance

In the current study, there was a sex tendency observed with steers tending to be heavier than heifers (*p* = 0.07). There was also an effect of sex on gain from pre-weaning to weaning (*p* = 0.02). This was to be expected as steers generally grow at a rate of 10–15% more than heifers [40] and have greater ADG [41]. Based on TRT, there were no differences in BW or gain for the duration of the study (Table 5).

These findings are consistent with previous research reporting limited effects of ITM administration on post-weaning performance. A meta-analysis by McKnight et al. [32] found that ITM administration did not impact cattle ADG. Similarly, Arthington et al. [42] observed no effect on overall ADG or BW gain in pre- and post-weaned calves. Additionally, Vedovatto et al. [43] reported no effect of ITM on post-weaning weight gain. In contrast, Bittar et al. [44] found that calves that received an ITM had greater ADG than calves that did not from D0 to D14. This is further supported by Mattioli et al. [45] who observed heavier BW at 30 d post-weaning under low-stress management conditions in heifer calves, although differences were not sustained to 60 d. In the current study, while treatment effects were not observed, all calves demonstrated the expected pattern of weight loss following stressors such as weaning, transport, and dietary transition [17,46,47], followed by gradual recovery. Under the management conditions of the current study, administration of ITM either prior to or at weaning did not influence overall growth performance.

Despite improvements in serum TM concentrations (discussed in greater detail in Section 3.2), observed in PW and WEAN calves, these changes did not translate into measurable differences in growth performance. This may indicate that, although ITM administration increased circulating mineral availability, baseline mineral status and overall nutritional management were sufficient to support growth across all treatment groups. This aligns with previous research showing that nutritional imbalances and diet composition can influence physiological responses and performance variability in young cattle [48]. Additionally, performance responses to TM supplementation are most often pronounced in cattle experiencing greater physiological stress, health challenges, or severe deficiencies. In the current study, the relatively low morbidity, absence of commingling, and controlled nutritional environment may have limited detectable performance responses to ITM.

#### 3.1.2. Dry Matter Intake and Shrink

Total dry matter intake (DMI) tended to be greater for the WEAN group throughout the post-weaning period (Table 6; *p* = 0.15) while feed DMI did not differ based on TRT (*p* = 0.44). These results partially align with the absence of TRT effects on BW and ADG.

Increased DMI following ITM administration has been reported in highly stressed, high-risk receiving calves [18,49]. Rauch et al. [50] found that calves that were two-step weaned and administered an ITM had greater DMI from D0 to D124 in comparison to calves that were abruptly weaned with an ITM injection and control calves. These findings suggest that weaning method in conjunction with ITM administration may influence calf DMI following weaning. However, several studies report that the utilization of ITM often does not affect intake directly unless there are underlying nutritional deficiencies present or health challenges [51,52]. Hong et al. [26] showed similar results with no differences in overall DMI in received feedlot cattle following an ITM. These results indicate that under the nutritional and management practices in this study, ITM timing had limited short-term impact on feed intake behavior.

In the current study, hay DMI tended to be higher (*p* = 0.06) in the WEAN group (1.80 kg) compared to the CON (1.56 kg) and PW groups (1.56 kg). This increase could potentially reflect a short-term behavioral or stress-mediated response following weaning and processing. Richeson and Kegley [18] observed increased DMI in received heifers with unknown backgrounds that were administered ITM. Berry et al. [49] reported increased feed intake from D29 to D42 with ADG and gain: feed (G:F) tended to improve for newly received calves given an ITM versus a negative control. However, those studies evaluated calves classified as high-risk and experiencing greater health challenges than calves in the current study. As total DMI and ADG did not differ between TRT groups in the current study, ITM timing appears to have had minimal impact on overall intake patterns under current study management conditions.

Calves experienced approximately 6% shrink (~14 kg) following weaning and simulated marketing. Shrink represents the combined effects of feed and water restriction, gut emptying, and tissue fluid losses, and is a common response to marketing and transport stress [6,11]. The magnitude of shrink observed in this study is within the range commonly reported for transported calves, which typically lose approximately 4% to 9% of BW depending on management and transport conditions [8,9]. In this study, shrink was compounded by the novelty of being subjected to simulated marketing conditions, the receiving environment, and dietary transition, thus resulting in calves requiring 21 d to surpass their weaning weight. This extended recovery period underscores the economic and welfare implications of stress-induced weight loss, as calves may lose valuable gain during the critical early backgrounding phase. Previous research supports these findings. Price et al. [10] evaluated post-weaning weight loss in calves, in which weight loss was exacerbated in the week following abrupt weaning. Calves that were abruptly weaned spent more time pacing compared to the control, as well as less time grazing or eating hay. Although Hersom et al. [53] did not evaluate the use of an ITM, their findings indicate that shrink is influenced not only by diet but also by handling environmental changes, and stress-related tissue losses.

#### 3.1.3. Adaptation to Target Intake and Potential Economic Implications

**Figure 2:**
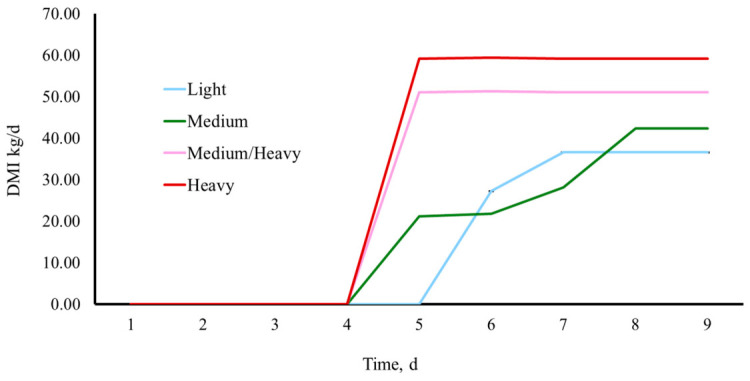
Days to reach targeted calf dry matter intake (DMI) by weight group (light, medium, medium/heavy, and heavy) between D1 and D9. Weights are based on a pen average by groups as follows: Light ≈ 194 kg; Medium ≈ 200 kg; Medium/Heavy ≈ 260 kg; Heavy ≈ 265 kg.

Reduced intake during the receiving period is well documented, with calves consuming approximately 1.5% of BW during the first 2 week after arrival [54,55]. Using ruminally cannulated calves, Fluharty et al. [56] determined that DMI was reduced 36% up to 7 d post weaning. In the current study, upon arrival at the stocker unit, calves were limit-fed at 2% BW with ad libitum access to hay. Medium/heavy and heavy calves (≈260–265 kg) reached target intake within approximately 4 d, whereas lighter calves (≈194–200 kg) required up to 8 d following weaning, processing, and transportation (Figure 2).

This delayed adaptation in lighter calves likely contributed to the prolonged weight recovery observed following shrink. Similarly, in high-risk cattle received on pasture, Rivera et al. [57] observed that it took these cattle up to 7 d to achieve targeted intake of a supplement offered to these calves. From an economic perspective, feed costs represent one of the largest expenses in preconditioning programs, typically accounting for 45–60% of the total budget [58]. When calves are limit-fed following weaning and transport but fail to consume the offered ration for several days, the cost of feed delivery is incurred without a return on animal performance. Additionally, delayed intake recovery may extend the number of days required to regain pre-shrink BW, increasing labor and overhead expenses on a per-calf basis. In the current study, as calves required up to 8 d to achieve target intake, it is possible that a substantial portion of feed resources may have been underutilized during this time. When evaluating BW and gain on the basis of TRT, it took 21 d to return to BW measured at weaning. This inefficiency, combined with shrink-related weight loss and delayed weight recovery, may compound the economic impact of post-weaning stress.

### 3.2. Serum Minerals

Serum trace minerals were evaluated to assess the effects of ITM administration on systemic mineral status during the weaning and post-weaning period. The primary minerals of interest were Cu, Se, Zn, and Mn, as these were included in the ITM formulation and play major roles within metabolism and immune function [39]. As previously mentioned, calves had ad libitum access to a complete mineral supplement prior to treatment allocation. Therefore, baseline serum mineral concentrations varied among individuals, with some animals falling below or near published adequacy ranges. This variability was accounted for within statistical models and may have influenced the magnitude of response to ITM administration. Additionally, although calves were limit-fed at 2.0% BW to standardize nutrient intake during the receiving period, this feeding strategy was consistent across all treatments and is not expected to have differentially influenced mineral metabolism or treatment responses.

#### 3.2.1. Copper (Cu)

Initial serum Cu concentrations measured on D-28 differed between treatments with WEAN calves having greater Cu concentrations (0.53 µg/mL) compared with PW (0.48 µg/mL) and CON (0.44 µg/mL) calves. Therefore D-28 serum Cu concentrations were used as a covariate for Cu analysis. Adjusted serum Cu concentrations are presented in Table 7.

Following adjustment, a TRT effect was observed (*p* = 0.02) with PW calves showing greater adjusted Cu concentrations (0.59 µg/mL) compared with WEAN (0.49 µg/mL) and CON calves (0.48 µg/mL). As the baseline Cu covariate was significant (*p* < 0.01), it is possible that initial Cu status at D-28 strongly influenced serum Cu concentrations at weaning (D0). These results indicate that administration of ITM 28 d prior to weaning increased serum Cu concentrations at D1 independent of baseline differences. In contrast, calves administered an ITM at D0 did not differ from the control group, likely reflecting the short interval (24 h) between injection and sample collection.

According to Herdt and Hoff [59], the reference range for serum Cu in growing cattle is 0.6–1.1 µg/mL. Based on this threshold, all calves were marginally deficient at the beginning of the study. Calves in the CON group remained below adequacy for the entirety of the study, whereas PW calves achieved marginal adequacy by D1 following ITM administration on D-28. Adequate Cu status prior to a stress event, such as weaning, may help preserve immune cell function by protecting neutrophils and macrophages from oxidative damage [25,60].

Although serum Cu concentrations increased in PW calves, circulating Cu responses to ITM administration are transient, peaking approximately 10–12 h post-injection and generally returning to baseline within 24 h [30,61]. Multiple studies have demonstrated that ITM administration increases hepatic Cu reserves even when serum concentrations remain unchanged [30,61,62]. The liver serves as the primary storage pool for Cu and maintains reserves necessary for metabolic and immune function [63]. While liver Cu concentrations were not evaluated in this study, greater serum Cu in PW calves at weaning suggests that pre-stress ITM administration improved systemic Cu availability during critical periods.

#### 3.2.2. Manganese (Mn)

Serum Mn concentrations prior to weaning (D-28) and at post-weaning and transport (D1) are presented in Table 7. There was no effect of TRT (*p* = 0.22), but there was an effect of time (*p* < 0.01) and a TRT × time interaction (*p* < 0.01) indicating that serum Mn concentrations increased over time in PW and WEAN calves. Serum Mn concentrations for the CON calves remained relatively the same at D-28 and D1. On D-28, prior to ITM administration, PW (3.70 ng/mL) and WEAN (3.50 ng/mL) calves had lower serum Mn concentrations compared to the CON group (4.90 ng/mL). Following ITM administration to the PW group on D-28, serum Mn concentrations on D1 increased to 4.93 ng/mL and WEAN calves to 7.16 ng/mL. Calves assigned to the CON treatment showed a slight increase to 5.12 ng/mL. In the current study, observed serum Mn values fell within adequate reference values of 0.9–6.0 ng/mL [59]. This suggests that ITM administration improved systemic Mn availability in calves with initially lower concentrations. Additionally, the observed increase in serum Mn in the WEAN group on D1 suggests that Mn from ITM administered at weaning is rapidly bioavailable and incorporated into circulation.

Research related to Mn storage and tissue distribution remains limited and reports variable responses to ITM administration [61]. Willmore et al. [64] observed reduced liver Mn concentrations in cows receiving ITM pre-calving and pre-breeding. In contrast, Pogge et al. [61] reported elevated plasma Mn concentrations for up to 15 d post-injection in newly weaned feedlot calves, with plasma Mn peaking at 8 and 10 h after injection before returning to baseline by D8 alongside increased liver Mn concentrations. Similarly, Arthington et al. [42] reported increased circulating Mn following ITM administration but did not show a consistent improvement in performance or other biological outcomes. However, Vedovatto et al. [30] found no effect of ITM administration on liver Mn concentrations at D0, D14, or D197 in weaned calves. These studies show that serum and tissue responses to ITM vary and may be dependent on timing of administration, animal age, and physiological status. Similar to Cu, liver Mn reserves may not always reflect systemic availability.

#### 3.2.3. Selenium (Se)

An effect of TRT (*p* < 0.01), time (*p* < 0.01), and their interaction (*p* < 0.01) was observed in serum Se (Table 7). Over time, serum Se concentrations increased in both PW and WEAN calves, whereas CON calves showed a numerical decrease from D-28 (23.3 ng/mL) to D1 (19.4 ng/mL). Despite increased serum Se concentrations in PW and WEAN calves, all calves in this study remained well below the reported adequacy range (65–140 ng/mL; [59]), indicating a persistent population-level Se deficiency that was not corrected by ITM administration within the study period despite increases in circulating Se. Additionally, these results suggest that while an ITM raised circulating Se concentrations, it was insufficient to fully correct marginal deficiency within the timeframe of this study. Previous research has demonstrated that ITM can increase Se stores in tissues such as the liver, even when serum concentrations remain low [64,65]. As liver Se was not assessed in the current study, it is possible that our results may not fully reflect total Se status or body reserves, particularly during periods of stress.

The observed increase in serum Se concentrations in the WEAN and PW groups suggest administration of an ITM 28 d prior to or at weaning may be an effective method for increasing Se serum concentrations compared to calves not receiving an ITM injection. Although serum Se remained below adequacy, ITM may enhance antioxidant capacity through Se-dependent enzymes such as glutathione peroxidase. Glutathione peroxidase functions to protect neutrophils and macrophages from oxidative damage and supports overall immune competence during periods of stress [66]. Administration of ITM at weaning could potentially contribute to resilience during weaning and the post-weaning periods. Practically, these findings suggest that in calves originating from marginal Se status herds, ITM alone may not be sufficient as a standalone strategy to achieve adequate Se status. Additional nutritional or management interventions may be required. Future research should evaluate whether ITM use can enhance Se-dependent enzyme activity or attenuate biological markers of stress and inflammation during weaning and transport.

#### 3.2.4. Zinc (Zn)

Zinc is an essential TM involved in numerous enzymatic reactions, immune function, and growth in cattle [59]. Metabolism of Zn can be influenced by dietary intake, stress, developmental stage, sex, and potentially breed [61,67,68]. In the current study, no TRT (*p* = 0.23), time (*p* = 0.83), or TRT × time interaction effects (*p* = 0.28) were observed. A sex effect was observed (*p* < 0.01), with heifers having greater overall Zn concentrations (0.84 µg/mL) compared with steers (0.78 µg/mL). Overall, results suggest that sex, rather than ITM administration or sampling time, was the primary driver of variation in serum Zn concentrations in this study. When evaluated further, tendencies were observed for sex x TRT (*p* = 0.08) and sex x time (*p* = 0.06; Table 8) interactions, indicating that calf response to ITM and weaning differed by sex.

Heifer calves showed numerically higher Zn concentrations compared with steer calves at both D-28 and D1. When assessing TRT, heifer calves assigned to the PW (0.86 µg/mL) and WEAN (0.86 µg/mL) groups maintained numerically greater serum Zn concentrations than steer calves in the same groups (PW, 0.73 µg/mL and WEAN, 0.81 µg/mL, respectively). Serum Zn remained relatively similar between steer and heifer calves that did not receive an ITM injection. Regardless of TRT, all calves had a marginally adequate serum Zn status as based on the reference range (0.6–1.9 µg/mL) suggested by Herdt and Hoff [59].

Little is known about the influence of sex on liver, plasma, and serum Zn concentrations in cattle and the research that is available shows varied results. Vedovatto et al. [30] showed no increase in serum Zn concentrations during a 51 d sampling period in both male and female calves following ITM administration. Although kidney samples were not collected in the current study, research conducted by Miranda et al. [69] found that kidney and blood Zn concentrations were significantly higher in females then males. However, calves in the aforementioned study ranged from 6 to 12 months of age with Zn concentrations not adjusted for the effects of age. Miranda et al. [69] speculated that sex-based differences could be linked to hormones (estrogen and testosterone) or the differences in growth rate between males and females, with males diverting more nutrients to tissue accretion. It should be noted that in the specified study, it was unknown if the male calves were castrated.

The sex-related differences observed in the current study may reflect a combination of developmental stage and growth dynamics rather than a direct treatment effect. Calves in the current study were weaned around 7 months of age, falling into the developmental stage where Zn concentration begins to decline compared to milk-fed calves. This could explain the slight decrease in heifer Zn concentrations on D1, although previous research has only evaluated age-related differences utilizing liver Zn concentrations [68]. In a study by Puschner et al. [68], liver Zn concentrations decreased as calves aged, stabilizing at the yearling or finishing stage, with differences observed in young calves (<3 months) and calves between 6 and 12 months of age. In the current study, heifers consistently had numerically greater serum Zn concentrations than steers at both sampling dates, and a tendency to have greater Zn concentrations following ITM administration. Zinn et al. [41] demonstrated that steers have greater growth rates (ADG) compared to heifers. The greater rate of gain exhibited by steers might result in a greater nutrient demand for Zn compared to heifers [70,71]. Further research is needed to better characterize sex- and age-related differences in Zn utilization during periods of stress and development.

### 3.3. Calf Health and Stress Parameters and Morbidity

Given the role of TMs in modulating stress physiology and immune function, their effects are commonly assessed using indicators such as antibody responses, haptoglobin, and cortisol. Additionally, the absence of commingling during simulated marketing may have reduced overall stress intensity compared with commercial conditions, which may have contributed to limited treatment differences.

#### 3.3.1. Bovine Respiratory Syncytial Virus (BRSV) Antibody Titers

Previous research has proposed that administration of an ITM can mediate animal stress responses by supporting immune function and antioxidant capacity [30,72], as well as increasing systemic mineral levels [61,64]. In particular, TMs such as Cu, Se, and Zn have been shown to have important roles in immune function support and antibody protection, especially under periods of heightened stress or mineral deficiency [24,73,74]. In the current study, BRSV antibody titers did not differ among treatment groups (*p* = 0.66; Table 9).

This indicates that ITM administration, whether 28 d prior to weaning or at weaning, did not influence the humoral immune response to BRSV vaccination during the receiving period and under the conditions of this study. The absence of differences in titer response could indicate adequate baseline mineral status or insufficient stressors to influence a differing response among treatments. This is also consistent with studies where ITMs had little influence under low-stress, well-managed conditions, suggesting that the benefit of an ITM on antibody response may be context-dependent and most evident in younger or more stressed calves [25,43]. As well as there being a lack of TRT differences, there was no TRT x time interaction (*p* = 0.76). However, there was an observed tendency of effect of time (*p* = 0.13). Although numerical increases in antibody titers were observed between D-28 and D0, these changes likely reflect a typical vaccine-induced primary and booster response. Additionally, BRSV titers remained within expected ranges following vaccination [72,75] suggesting that calves were able to produce a consistent immune response regardless of ITM administration timing.

Previous studies have reported improved antibody responses with the use of ITM in conjunction with BRD vaccination, although outcomes often depend on calf age, baseline TM status, environmental challenges, and timing of ITM administration. Bittar et al. [44] reported that ITM administered with a BRD vaccination improved immune responses to *M. haemolytica* and *P. multocida* in 3-month-old dairy calves. This immune response in young calves suggests that the use of ITM is beneficial to the calf surmounting an effective antibody response to vaccination. In feedlot cattle, ITM did not improve performance or morbidity; however, bovine viral diarrhea virus (BVDV)-specific antibody responses were higher at D14 in ITM-treated groups [52]. Similarly, Arthington and Havenga [51] demonstrated that ITM administration in newly received beef calves (10–11-month-old) alongside BRD MLV may enhance the production of neutralizing antibodies to bovine herpesvirus-1 (BHV-1). Conversely, Palomares et al. [76] found no significant effect of ITM on antibody titers in 3-month-old dairy calves when ITM was administered at the time of BRD vaccination, with no increase in BRSV or BHV-1 titers observed. This is further supported by dos Reis et al. [29] who found no impacts of ITM administration on BRSV titers of weaned calves that either received an estradiol implant or no implant.

#### 3.3.2. Serum Haptoglobin

Haptoglobin is an acute phase protein (APP) that increases in response to systemic inflammation and physiological stress in cattle, particularly during events such as weaning, transportation, and commingling [27,76,77]. In the current study, serum haptoglobin concentrations did not differ based on TRT (*p* = 0.99) nor was there a TRT × time interaction effect observed (*p* = 0.30; Table 9). Overall mean haptoglobin values were similar among groups, averaging 4.14 ng/mL (CON), 4.12 ng/mL (PW), and 4.12 ng/mL (WEAN), respectively. Across all treatments, serum haptoglobin increased shortly after weaning and simulated marketing, reflecting an effect of time (*p* < 0.01). The CON and PW groups showed a decrease in haptoglobin by D7. However, haptoglobin slightly increased in the WEAN group at D7.

The observed increased haptoglobin concentrations following weaning and transportation align with the expected acute phase response to physiological stress in beef calves [27,77]. Haptoglobin is commonly used as a biomarker of systemic inflammation during the post-weaning transition with the observed response in this study aligning with that of previous studies [77,78]. Studies have also shown that prolonged acute phase responses, including sustained haptoglobin levels, are negatively correlated with performance in healthy cattle [79,80]. In the current study, however, haptoglobin concentrations were only assessed through D7; extending measurements beyond this point could provide greater insight into the duration of the inflammatory response and its potential implications for performance.

There is a known role of Cu, Se, and Zn in mitigation of oxidative stress and inflammation [25]. Previous research has shown that the use of ITM may reduce systemic inflammatory and APP expression and responses in calves experiencing significant immune or environmental challenges [26,30]. In the current study, the use of ITMs, either 28 d prior to weaning or at weaning, did not influence calf serum haptoglobin concentrations. While all calves exhibited the expected physiological responses to weaning and transportation, the absence of treatment differences suggests that ITMs did not significantly modify this response under the conditions of this study. The lack of response in calves could reflect adequate baseline nutrition and mineral status, which is supported by Willmore et al. [64]. Although haptoglobin was not evaluated, Willmore et al. [64] found that while ITM administration improved liver TM status, it did not improve calf pre-weaning growth, likely because baseline mineral status was adequate in a non-stressed herd. This aligns with calf performance results of this study. Further evaluation of calves in a mineral-deficient status or in a more severely challenged population under different management practices may help clarify the role of ITM in modulating inflammatory responses during the post-weaning period.

#### 3.3.3. Hair Cortisol Concentrations

Hair cortisol concentration (HCC) provides a retrospective measure of hypothalamic–pituitary–adrenal (HPA) axis activity and can be used as a biomarker of chronic stress [36,81]. Hair cortisol concentrations measured at D0 and D42 are represented in Table 9. No differences were observed among TRT groups (*p* = 0.20) nor was there a TRT x time effect (*p* = 0.86). However, there was a time effect (*p* < 0.01) with all treatments showing an increase in cortisol concentration between D0 and D42. Prior to weaning and transport (D0), HCC ranged from 1.55 pg/mg (WEAN) to 1.83 pg/mg (PW). Following weaning and transportation (D42), HCC increased across all groups ranging from 2.70 pg/mg (WEAN) to 3.13 pg/mg (PW). This increase in HCC is consistent with the accumulation of cortisol in the hair shaft as a response to stress over an extended period and aligns with the expected physiological stress of a weaning and post-weaning transition period [82].

Previous studies evaluating cortisol responses to weaning and stressors in calves have primarily relied on plasma, serum, or salivary cortisol measurements. Mac et al. [3] investigated salivary cortisol concentrations following weaning in calves and found that following a stress event, there was a salivary cortisol concentration increase up to 14 d following weaning. Campistol et al. [83] assessed total plasma cortisol levels following weaning, in which two different weaning methods were used. Calves that were abruptly weaned showed an increase in plasma cortisol concentrations at D3. Similarly, Kim et al. [84] reported increased serum cortisol concentrations after weaning. Within these studies, plasma and blood cortisol concentrations increased at weaning or following stressful events. However, it should be noted that plasma, serum, and salivary cortisol concentrations provide an accurate representation of an animal’s stress level at a given time point [85], whereas HCC reflects long-term changes associated with chronic stress. Hair cortisol may be more suitable for documenting overall stress load rather than detecting short-term or subtle changes. Future work that integrates both long-term (HCC) and acute measures of cortisol (plasma or salivary) could provide greater clarity on whether ITM administration primarily influences immediate stress responsiveness, longer term adaptation, or both.

#### 3.3.4. Calf Morbidity

Calf morbidity did not differ among treatments (*p* = 0.34) with morbidity rates at 7.7% (CON), 17.3% (PW), and 19.2% (WEAN), respectively. However, the low incidence of morbidity may have limited the statistical power to detect treatment differences. Additionally, mortality was minimal across all groups. This is consistent with a meta-analysis by McKnight et al. [32] who found that ITM administration did not affect overall morbidity outcomes within preconditioning and feedlot-receiving phases. Nevertheless, data exist demonstrating that ITM administration can improve the health of high-risk newly received cattle [18]. It should be noted that this inconsistent response in the literature may be due to the type of cattle used and based on their prior management conditions.

As discussed previously, there were no treatment effects observed for BRSV antibody titers. However, titers increased following vaccination, indicating that calves mounted an appropriate immune response regardless of ITM administration. These findings suggest that ITM timing did not influence morbidity or antibody response under the conditions of this study. In the present study, cattle would be considered “low risk” as they were from a single, well-managed herd, with consistent nutrition and health management strategies. The overall low morbidity and adequate vaccination response may indicate that calves were not experiencing sufficient mineral deficiencies or health challenges to elicit a measurable benefit from ITM administration. However, in the current study, morbidity was only assessed for animals exhibiting clinical signs of illness. Infections with BHV-1 and BVDV may occur in the absence of clinical symptoms, potentially altering health outcome data [18,86,87,88].

### 3.4. Study Limitations and Implications

The limited effects of ITM administration observed in this study should be interpreted in the context of the relatively low-stress and well-managed conditions under which calves were evaluated. Calves originated from a single herd with prior access to a complete mineral supplement. Additionally, they were not commingled with unfamiliar animals and experienced a relatively short transport distance (9.7 km) under controlled receiving conditions. These conditions likely reduced overall physiological stress and contributed to adequate baseline TM status. Under these conditions, benefits from ITM administrations at or prior to weaning were minimal. From a practical standpoint, these findings suggest that ITM administration in similarly managed, low-risk herds may provide limited return on investment, although further research is needed. Administration of ITM may be more effectively targeted towards calves originating from higher-risk sources, stressful marketing environments, or those with greater dietary or mineral uncertainty where physiological demand for TM is expected to be greater. Future studies could be enhanced by integrating precision livestock tools to continuously monitor behavior and physiological responses as these could capture more transient treatment effects that may not be detectable through periodic sampling alone.

## 4. Conclusions

This study evaluated the effects of ITM administration either 28 d prior to weaning or at weaning in conjunction with simulated marketing on stress biomarkers, mineral status, immune response, and performance in beef calves. Under the conditions of a well-managed herd, ITM timing did not alter antibody titer response to vaccination, long-term cortisol accumulation, morbidity, or overall performance outcomes. These results suggest that the humoral immune response and systemic inflammatory responses were largely maintained regardless of ITM administration strategy. Additionally, these findings indicate that ITM administration can improve systemic TM concentrations during the weaning and receiving period. However, its influence on immune response, stress physiology and growth performance may be context dependent. In environments where calves are managed under low-stress conditions with marginal to adequate mineral status, the benefits of ITM on health and performance appear limited. Future research should focus on populations exposed to greater nutritional challenges, heightened stress, extended transportation, or increased disease risk, where strategic ITM administration may play a more critical role in supporting immune competence and animal productivity.

## Figures and Tables

**Table 1:** Composition of loose mineral offered to dams and calves prior to the treatment period.

Item	Inclusion Level
Ca ^1^, %	15.0–18.0
P ^2^, %	6.00
Salt, %	16.0–19.2
Mg ^3^, %	5.00
Mn ^4^, ppm	1000
Zn ^5^, ppm	3000
Cu ^6^, ppm	1250
Se ^7^, ppm	20.0
Co ^8^, ppm	20.0
Iodine, ppm	25.0
Vitamin A, IU/kg	661,000
Vitamin D, IU/kg	66,000
Vitamin E, IU/kg	220

^1^ Calcium. ^2^ Phosphorous. ^3^ Magnesium. ^4^ Manganese. ^5^ Zinc. ^6^ Copper. ^7^ Selenium. ^8^ Cobalt.

**Table 2:** Composition of injectable trace mineral (ITM; Multimin 90) injection administered to calves 28 d prior to weaning (PW calves) and at weaning (WEAN calves).

**Active Ingredients**	**Concentration, mg/mL**	**Form**
Cu ^1^	15.0	as copper carbonate
Mn ^2^	10.0	as manganese carbonate
Se ^3^	5.0	as sodium selenite
Zn ^4^	60.0	as zinc oxide
**Inactive Ingredients**	**Concentration, mg/mL**	**Function**
Eidetic (EDTA) acid	399.7	chelating agent
Sodium hydroxide	106.9	-
Benzyl alcohol	10.4	preservative

^1^ Copper; ^2^ Manganese; ^3^ Selenium; ^4^ Zinc.

**Table 3:** Ingredient composition of starter ration provided to calves during the post-weaning receiving period.

Ingredient	Inclusion, % DM ^1^
Soybean hull pellets	35.3
Corn gluten feed	25.2
Dried distiller’s grain (corn)	12.1
Beet pulp pellets	12.2
Cottonseed hulls	7.06
Sunflower meal	3.53
Mineral package	1.81
Conditioner	1.82
Limestone	0.48
LNC 5 Rumensin pellet ^2^	0.26
Salt	0.18

^1^ Dry matter; ^2^ Livestock Nutrition Center; 130 g monensin/ton.

**Table 4:** Nutrient composition of annual ryegrass (*Lolium multiflorum* Lam.) hay and starter ration provided to calves during the 42 d post-weaning period.

Item	Starter Ration	Ryegrass Hay
DM ^1^, %	91.2	91.9
CP ^2^, % DM	15.7	17.8
ADF ^3^, % DM	30.8	38.8
aNDF ^4^, % DM	47.0	64.7
TDN ^5^, % DM	72.0	56.0
NE_m_ ^6^, Mcal/kg	0.35	0.22
NE_g_ ^7^, Mcal/kg	0.22	0.11
Ca ^8^, %	1.16	0.77
P ^9^, %	0.48	0.34
Mg ^10^, %	0.34	0.45
K ^11^, %	1.39	0.90
Na ^12^, %	0.22	0.29
Fe ^13^, ppm	571.0	274.0
Zn ^14^, ppm	116.0	47.0
Cu ^15^, ppm	23.0	20.0
Mn ^16^, ppm	66.0	112.0
Mo ^17^, ppm	0.50	1.60

^1^ Dry matter. ^2^ Crude protein. ^3^ Acid detergent fiber. ^4^ Amylase-treated neutral detergent fiber. ^5^ Total digestible nutrients. ^6^ Net energy of maintenance. ^7^ Net energy of gain. ^8^ Calcium. ^9^ Phosphorous. ^10^ Magnesium. ^11^ Potassium. ^12^ Sodium. ^13^ Iron. ^14^ Zinc. ^15^ Copper. ^16^ Manganese. ^17^ Molybdenum.

**Table 5:** Effects of injectable trace mineral (ITM) administration on performance of calves receiving an ITM 28 d prior to weaning (D-28) or at weaning (D0).

	Treatments ^1^				
Item	CON	PW	WEAN	SEM	*p*-Value
Weight, kg					
Pre-weaning (D-28)	224.5	222.5	225.4	19.8	0.95
Weaning (D0)	237.7	234.7	236.8	20.4	0.94
D1	222.7	221.1	222.4	19.4	0.98
D7	228.2	228.5	224.8	19.9	0.90
D21	239.2	237.0	237.7	20.3	0.97
D42	257.9	254.3	256.4	20.2	0.92
Gain, kg					
Weaning (D0)	13.2	12.2	11.4	3.16	0.47
D1	−14.9	−13.6	−14.5	4.49	0.79
D7	−9.5	−6.2	−12.1	7.41	0.21
D21	1.6	2.3	0.9	4.45	0.78
D42	20.4	19.6	19.6	5.20	0.94

^1^ Treatments were as follows: (1) no injectable trace mineral (ITM; CON), (2) subcutaneous ITM administration 28 d prior to weaning (PW), or (3) ITM administration on the day of weaning (WEAN).

**Table 6:** Effects of injectable trace mineral (ITM) administration on intake of calves receiving an ITM at pre-weaning or at weaning.

	Treatments ^1^				
Item, kg/d	CON	PW	WEAN	SEM	*p*-Value
Feed DMI ^2^	3.27	3.15	3.26	0.21	0.44
Hay DMI	1.56	1.56	1.80	0.20	0.06
Total DMI	4.84	4.72	5.06	0.33	0.15

^1^ Treatments were as follows: (1) no injectable trace mineral (ITM; CON), (2) subcutaneous ITM administration 28 d prior to weaning (PW), or (3) ITM administration on the day of weaning (WEAN). ^2^ Dry matter intake.

**Table 7:** Effects of timing of administration of an injectable trace mineral (ITM) on serum mineral concentrations prior to weaning (D-28) and post-weaning (D1).

	Treatments ^1^				*p*-Value		
Serum Mineral	CON	PW	WEAN	SEM	TRT ^2^	Time	TRT × Time
Copper (Cu), µg/mL ^3^	0.48 ^b^	0.59 ^a^	0.49 ^b^	0.03	0.02	-	-
Manganese (Mn), ng/mL ^4^				0.58	0.22	<0.01	<0.01
D-28 ^5^	4.90 ^a^	3.70 ^b^	3.50 ^b^				
D1 ^6^	5.12 ^b^	4.93 ^b^	7.16 ^a^				
Selenium (Se), ng/mL				2.62	<0.01	<0.01	<0.01
D-28	23.3	25.3	26.0				
D1	19.4 ^c^	41.4 ^a^	30.9 ^b^				

^1^ Treatments were as follows: (1) no injectable trace mineral (ITM; CON), (2) subcutaneous ITM administration 28 d prior to weaning (PW), or (3) ITM administration on the day of weaning (WEAN). ^2^ TRT: Treatment. ^3^ A significant TRT effect was observed for baseline serum Cu concentrations collected on D-28; therefore, initial serum Cu was used a covariate in the subsequent analysis. ^4^ For Mn and Se, *p*-values represent overall model effects of TRT, time, and TRT × time from repeated-measures analysis. ^5^ D-28: 28 d prior to weaning. ^6^ D1: 1 d post-weaning and simulated marketing. ^abc^ Means within row differ (*p* ≤ 0.05).

**Table 8:** Effects of timing of administration of an injectable trace mineral (ITM) on serum zinc (Zn) concentrations based on sex and its interactions with treatment (TRT) and time.

	Treatments ^1^							*p*-Value
**Zinc (Zn), µg/mL**	**CON**		**PW**		**WEAN**		**SEM**	**Sex × TRT ^2^**
Sex ^3^							0.03	0.08
Steer	0.79		0.73		0.81			
Heifer	0.79		0.86		0.86			
**Item**	**Sex**						**SEM**	**Sex × Time**
Time ^4^	Steer			Heifer			0.02	0.06
D-28 ^5^	0.76			0.85				
D1 ^6^	0.80			0.82				

^1^ Treatments were as follows: (1) no injectable trace mineral (ITM; CON), (2) subcutaneous ITM administration 28 d prior to weaning (PW), or (3) ITM administration on the d of weaning (WEAN). ^2^ TRT: Treatment. ^3^ *p*-values represent overall model effects of sex × TRT. ^4^ *p*-values represent overall model effects of sex × time. ^5^ D-28: 28 d prior to weaning. ^6^ D1: 1 d post-weaning and simulated marketing.

**Table 9:** Effects of timing of administration of an injectable trace mineral (ITM) on calf health and stress biomarkers.

	Treatments ^1^				*p*-Value ^2^		
Item	CON	PW	WEAN	SEM	TRT ^2^	Time	TRT × Time
BRSV SN, log_10_^3^				0.10	0.66	0.13	0.76
D-28	0.35	0.45	0.35				
D0	0.65	0.50	0.50				
D21	0.50	0.55	0.45				
Haptoglobin, log(HP), ng/mL ^4^				0.17	0.99	<0.01	0.30
D0	3.22	3.15	3.29				
D1	4.76	4.63	4.39				
D7	4.45	4.59	4.70				
Hair Cortisol, pg/mg ^5^				0.17	0.20	<0.01	0.86
D0	1.63	1.83	1.55				
D42	2.89	3.13	2.70				

^1^ Treatments were as follows: (1) no injectable trace mineral (ITM; CON), (2) subcutaneous ITM administration 28 d prior to weaning (PW), or (3) ITM administration on the day of weaning (WEAN). ^2^ *p*-values represent overall model effects of TRT, time, and TRT × time. ^3^ Serum samples for bovine respiratory syncytial virus (BRSV) serum neutralizing (SN) antibody titer responses were collected on D-28 (28 d prior to weaning), D0 (weaning), and D21 (21 d post-weaning). ^4^ Serum samples for haptoglobin were collected on D0 (weaning), D1 (post-transport), and D7 (7 d post-weaning). ^5^ Samples for hair cortisol concentrations (HCC) were collected on D0 (weaning) and D42 (42 d post-weaning). Hair cortisol concentrations are based on pg of cortisol/mg of hair.

## Data Availability

Data that supports the findings of this study are available from the authors upon request.

## Abbreviations

The following abbreviations, in alphabetical order, are used in this manuscript:

ADF	Acid detergent fiber
ADG	Average daily gain
aNDF	Amylase-treated neutral detergent fiber
APP	Acute phase protein
BHV-1	Bovine herpesvirus
BRD	Bovine respiratory disease
BRSV	Bovine respiratory syncytial virus
BVDV	Bovine viral diarrhea virus
BW	Body weight
CON	Control treatment; did not receive an injectable trace mineral
CP	Crude protein
Cu	Copper
d	Day(s)
DM	Dry matter
DMI	Dry matter intake
ELISA	Enzyme-linked immunosorbent assay
G:F	Gain to feed ratio
h	Hour(s)
HCC	Hair cortisol concentration
HPA	Hypothalamic–pituitary axis
IACUC	Institutional Animal Care and Use Committee
ICP-MS	Inductively coupled plasma mass spectrometry
ITM	Injectable trace mineral
LNC	Livestock Nutrition Center
Mn	Manganese
Mn-SOD	Manganese superoxide dismutase
NE_g_	Net energy of gain
NE_m_	Net energy of maintenance
OTMS	Oral trace mineral supplement
PW	Pre-weaning treatment; calves received an ITM 28 d prior to weaning
RIA	Radioimmunoassay
Se	Selenium
SN	Serum neutralizing
SWREC	Southwest Research and Extension Center
TDN	Total digestible nutrients
TM	Trace mineral
TRT	Treatment
WEAN	Weaning treatment; calves received an ITM at weaning
yr	Year(s)
Zn	Zinc
